# Supplementary material for: High-resolution two-dimensional electronic spectroscopy reveals the homogeneous line profile of chromophores solvated in nanoclusters
Source: Nat Commun. 2022 Jun 10;13:3350. doi: 10.1038/s41467-022-31021-z (PMC9187667; doi:10.1038/s41467-022-31021-z)
Supplement: Supplementary file 1 — Supplementary information [file 41467_2022_31021_MOESM1_ESM.pdf]

Supplementary information: High-resolution two-dimensional electronic spectroscopy reveals the homogeneous line profile of chromophores solvated in nanoclusters

Ulrich Bangert[1], Frank Stienkemeier[1], and Lukas Bruder[1]*

[1]*Institute of Physics, University of Freiburg,*

Hermann-Herder-Str. 3, 79104 Freiburg, Germany

**lukas.bruder@physik.uni-freiburg.de*

(Dated: May 23, 2022)

I. SUPPLEMENTARY NOTE 1

Fig. 1 shows a zoom of the Fourier spectra from the 1D coherence scans of H_2Pc–He_N and H_2Pc–Ne_N (cf. Fig. 2b in the main text) along with excitation spectra obtained by light-induced fluorescence (LIF) measurements using continuous wave (cw) and nanosecond lasers. These spectra feature very narrow excitation bands and a distinct blue-shift compared to H_2Pc spectra in solution (Fig. 2b in the main text) or of hot H_2Pc molecules in the gas phase [1]. Due to the low internal temperature of the molecules in the cluster-isolation experiments only the vibrational ground state of H_2Pc is populated, which strongly curtails the number of possible vibronic transitions and reduces the excitation spectrum mainly to the ZPL of the Q_x absorption band. Due to the weakly perturbing cluster environment, the ZPL is hardly red shifted ($\leq 100\,\mathrm{cm}^{-1}$) with respect to the spectrum of cold gas phase molecules: ZPL in H_2Pc–He_N is at $15088.9\,\mathrm{cm}^{-1}$, in H_2Pc–Ne_N at $15032\,\mathrm{cm}^{-1}$ in in jet-cooled H_2Pc at $15132\,\mathrm{cm}^{-1}$ [2], hence, explaining the strong blue shift compared to experiments in solution.

In the H_2Pc–He_N data, the ZPL features a spectral wing on the high-frequency side, which shows no correlation with the ZPL [3] and was identified as a C_{13}-isotope of H_2Pc (ZPL at $15089.5\,\mathrm{cm}^{-1}$) [4]. In the H_2Pc–Ne_N spectrum this feature is obscured by the inhomogeneous broadening. Moreover, the phonon wing, a spectral feature attributed to the excitation of volume vibrations of the helium environment, appears weakly at approximately $15093\,\mathrm{cm}^{-1}$. However, its relative amplitude of 1 % is close to the SNR of the 1D coherence scan. Other resonances are not observed in the H_2Pc–He_N spectra at the low laser intensities. Only at two orders of magnitude higher laser intensities strongly saturating the ZPL, a weak coupling to vibrational modes of the molecule has been observed in the steady-state LIF measurements [4]. Likewise, in H_2Pc–Ne_N a weak vibronic coupling is only observed in the nanosecond laser experiment, where the optical transition was strongly saturated to enhance weak features.

II. SUPPLEMENTARY NOTE 2

In 2DES, the spectral line profile along the diagonal(anti-diagonal) correspond to the inhomogeneous(homogeneous) absorption profiles of the system [6]. To extract both

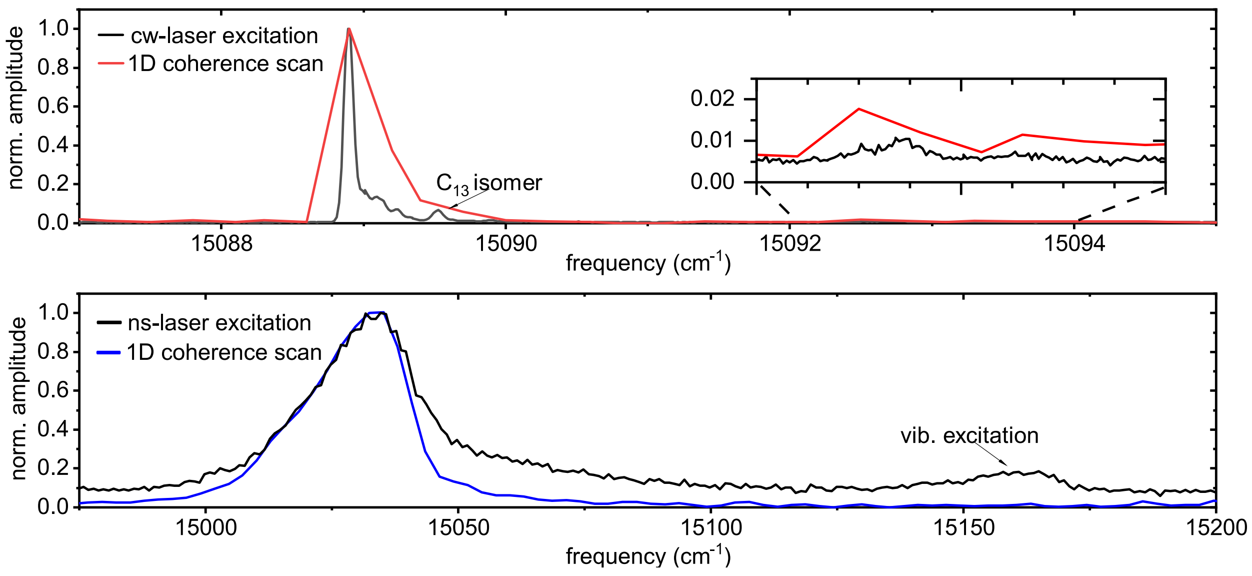

Supplementary Figure 1. Fourier spectra of 1D coherence scans of H_2Pc–He_N (red) and H_2Pc–Ne_N (blue) compared to LIF excitation spectra using narrow-band cw and nanosecond lasers (black). The inset shows the phonon wing. The reference spectrum of H_2Pc–He_N was taken from Ref. [3] and the one of H_2Pc–Ne_N was recorded in our laboratory with the experimental setup described in Ref. [5] at an oven cell temperature of 390 °C and laser pulse energy of 109 µJ. The high pulse energy in the latter experiment leads to saturation broadening of the strong absorption lines and a relative enhancement of the weak vibronic lines at 15160 cm^{-1}.

linewidths, we performed a 2D peak fit of the H_2Pc–Ne_N spectrum from Fig. 3c in the main text. To this end, we adapted the 2D fit function calculated by Bell et al. [7] and fitted the rephasing part of the 2D spectrum. In the homogeneously broadened dimension the fit function consists of a single Lorentzian profile, in the inhomogeneously broadened dimension we apply a sum of two Gaussian functions to account for the double peak structure (main peak and red shoulder) of the inhomogeneous profile, leading to the 2D line shape function:

$$
\begin{aligned}
S_R(\omega_t,\omega_\tau) = \sum_i^{1,2} A_i \frac{1}{2\sigma_i(2\gamma - i(\omega_t+\omega_\tau))} \\
\times [e^{\frac{(\gamma - i(\omega_t-\omega_{0i}))^2}{2\sigma_i^2}} \mathrm{Erfc}(\frac{\gamma - i(\omega_t-\omega_{0i})}{\sqrt{2}\sigma_i}) \\
+ e^{\frac{(\gamma - i(-\omega_\tau+\omega_{0i}))^2}{2\sigma_i^2}} \mathrm{Erfc}(\frac{\gamma - i(-\omega_\tau+\omega_{0i})}{\sqrt{2}\sigma_i})] \,.
\end{aligned} \tag{1}
$$

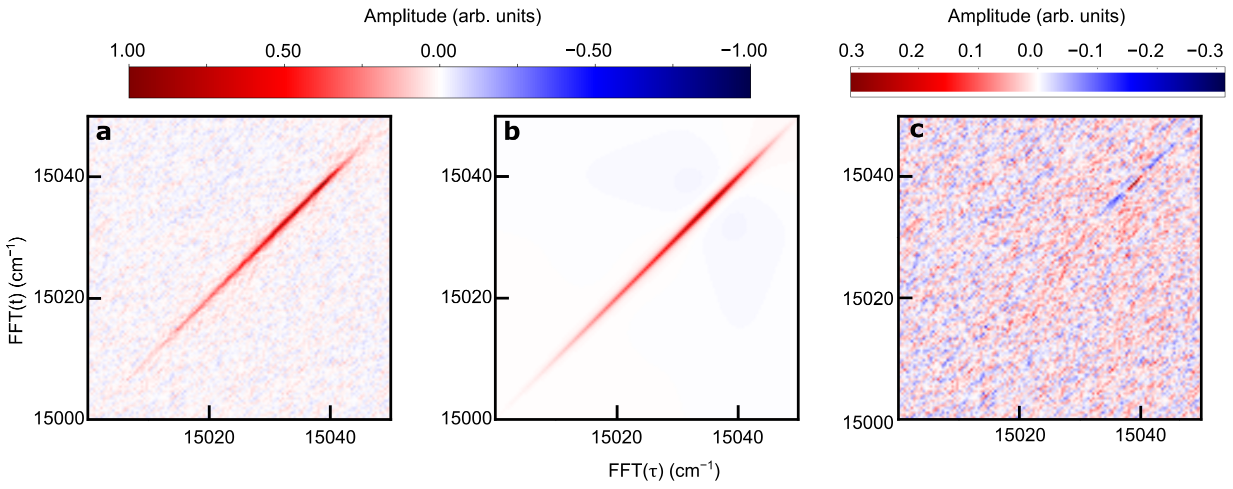

Supplementary Figure 2. 2D peak fit. (a) experimental H_2Pc–Ne_N data, (b) fitted function, (c) residues between (a) and (b).

Here A_i, ω_{0i} and σ_{0i} are the amplitudes, center frequencies and widths of the Gaussians. γ is the dephasing rate which is connected to the homogeneous line width by FWHM $= 2\gamma$. The 2D fit result is shown in Fig. 2 along with the residues. Except for an outlier in the experimental data at 15040 cm^{-1}, we get an excellent agreement between the experimental data and the fit result as indicated by the residues. The homogeneous line width is deduced directly from the 2D fit. The inhomogeneous line width is evaluated from the FWHM value of the 1D coherence scan.

III. SUPPLEMENTARY NOTE 3

To evaluate the number of binding configurations in the H_2Pc–Ne_N system, we fitted the inhomogeneous line profile of the 1D coherence scan (Fig. 1b) with the simplistic fit model described in the main text. For the fit, we consider the spectral interval 14980-15070cm^{-1}, where the H_2Pc–Ne_N spectrum shows a significant amplitude. The fit yields a minimum density of homogeneous absorption lines of $\geq$ 4 lines/cm^{-1}, amounting to a total number of 360 binding configurations. As the transition frequencies of the individual binding configurations are not likely distributed equidistantly, an even higher line density and, consequently, a higher number of configurations should be taken into account. On the other hand, the finite spectral resolution of the experiment introduces an uncertainty towards lower numbers of binding configurations. Since we measured the line profile in

the time domain over a finite time window (3 ps), a small uncertainty remains that the line profile exhibits a spectral substructure which is neither resolved in our experiment nor in the previous nanosecond laser LIF experiments. A sub-structure would lead to a lower line density required for the fitting of the data. Taking this factor into account, we compute an estimate for the lower limit of the number of binding configurations of ≥ 216, which corresponds to a mean energetic separation of the binding configurations of $0.42\,\mathrm{cm}^{-1}$. In principle, the C_{13}-isotopes of H_2Pc should be also taken into account for the fit procedure. However, their small spectral amplitudes ($\leq 10\%$ of ZPL) should make a negligible contribution and were therefore not considered.

Intuitively, the high number of configurations could be explained with the intrinsic properties of the neon clusters and the doping process. The broad distribution of cluster sizes and the icosahedral structure of the clusters form a large variety of slightly different surfaces the H_2Pc can attach to. In the nanosecond laser experiments (Fig. 1b) much larger clusters were used and essentially the same inhomogeneous line profile has been obtained. Likewise, in similar studies using PTCDA molecules no significant effect of the cluster size on the inhomogeneous line profile was found [5]. From this, we conclude, that the statistical cluster size distribution has a minor impact on the molecule-surface binding configurations and surface defects of the icosahedral geometry play a larger role. Further, each surface can have local potential minima depending on the orientation and position of the molecule. The molecules are likely trapped in these local minima, due to the random pick-up process and the rapid evaporative cooling afterwards.

SUPPLEMENTARY REFERENCES

[1] D. Eastwood, L. Edwards, M. Gouterman, and J. Steinfeld, Spectra of porphyrins: Part VII. Vapor absorption and emission of phthalocyanines, Journal of Molecular Spectroscopy **20**, 381 (1966).

[2] P. S. H. Fitch, C. A. Haynam, and D. H. Levy, The fluorescence excitation spectrum of free base phthalocyanine cooled in a supersonic free jet, J. Chem. Phys. **73**, 1064 (1980).

[3] R. Lehnig, J. A. Sebree, and A. Slenczka, Structure and dynamics of phthalocyanine- argon n (n= 1- 4) complexes studied in helium nanodroplets, The Journal of Physical Chemistry A **111**, 7576 (2007).

[4] R. Lehnig, M. Slipchenko, S. Kuma, T. Momose, B. Sartakov, and A. Vilesov, Fine structure of the (S1←S0) band origins of phthalocyanine molecules in helium droplets, J. Chem. Phys. **121**, 9396 (2004).

[5] M. Dvorak, M. Müller, T. Knoblauch, O. Bünermann, A. Rydlo, S. Minniberger, W. Harbich, and F. Stienkemeier, Spectroscopy of 3, 4, 9, 10-perylenetetracarboxylic dianhydride (PTCDA) attached to rare gas samples: Clusters vs. bulk matrices. I. Absorption spectroscopy, J. Chem. Phys. **137**, 164301 (2012).

[6] D. M. Jonas, Two-Dimensional Femtosecond Spectroscopy, Annu. Rev. Phys. Chem. **54**, 425 (2003).

[7] J. D. Bell, R. Conrad, and M. E. Siemens, Analytical calculation of two-dimensional spectra, Opt. Lett. **40**, 1157 (2015).
